# Comparative Study of the Antioxidant Constituents, Activities and the GC-MS Quantification and Identification of Fatty Acids of Four Selected *Helichrysum* Species

**DOI:** 10.3390/plants11080998

**Published:** 2022-04-07

**Authors:** Kolajo Adedamola Akinyede, Gail Denise Hughes, Okobi Eko Ekpo, Oluwafemi Omoniyi Oguntibeju

**Affiliations:** 1 Department of Medical Bioscience, University of the Western Cape, Bellville, Cape Town 7530, South Africa; ghughes@uwc.ac.za (G.D.H.); okobi.ekpo@ku.ac.ae (O.E.E.); 2 Department of Science Technology, Biochemistry Unit, The Federal Polytechnic P.M.B.5351, Ado Ekiti 360231, Nigeria; 3 Department of Anatomy and Cellular Biology, College of Medicine and Health Sciences, Khalifa University, Abu Dhabi P.O. Box 127788, United Arab Emirates; 4 Phytomedicine and Phytochemistry Group, Department of Biomedical Sciences, Faculty of Health and Wellness Sciences, Cape Peninsula University of Technology, P.O. Box 1906, Bellville, Cape Town 7535, South Africa

**Keywords:** helichrysum, antioxidant, fatty acids, drug discovery and development, DPPH radical scavenging, total phenolics

## Abstract

*Helichrysum* Mill. (Asteraceae) is a plant genus comprising distinctively of aromatic plants of about 500–600 species. Since most of these plants have not been previously studied, extensive profiling helps to validate their folkloric uses and determine their potential value as sources of plant-derived drug candidates. This study, therefore, aims to investigate the antioxidant activity (DPPH, NO, FRAP); total antioxidant capacity, total phenolic, total flavonoid, and fatty acid compositions of the aqueous acetone extracts from four *Helichrysum* plants namely, *Helichrysum pandurifolium, Helichrysum foetidum, Helichrysum petiolare*, and *Helichrysum cymocum*. The results obtained showed that the *H. cymocum* extract had the best DPPH radical scavenging activity (IC_50_ = 11.85 ± 3.20 µg/mL) and *H. petiolare* extract had the best nitric oxide scavenging activity (IC_50_ = 20.81 ± 3.73 µg/mL), while *H. pandurifolium* Schrank extract (0.636 ± 0.005 µg/mL) demonstrated the best ferrous reducing power, all of which are comparable with results from ascorbic acid used as the standard. The IC_50_ values of the radical scavenging activity ranged from 11.85–41.13 µg/mL (DPPH), 20.81–36.19 µg/mL (NO), and 0.505–0.636 µg/mL (FRAP), for all the plants studied. The *H. petiolare* has the highest total antioxidant capacity (48.50 ± 1.55 mg/g), highest total phenolic content (54.69 ± 0.23 mg/g), and highest total flavonoid content (56.19 ± 1.01 mg/g) compared with other species. The fatty acid methyl esters were analysed using gas chromatography-mass spectrometry (GC-MS). The results obtained showed variations in the fatty acid composition of the plant extracts, with *H. petiolare* having the highest saturated fatty acid (SFA) content (7184 µg/g) and polyunsaturated fatty acid (PUFA) content (7005.5 µg/g). In addition, *H. foetidum* had the highest monounsaturated fatty acid (MUFA) content (1150.3 µg/g), while *H. cymocum* had the highest PUFA:SFA ratio of 1.202. In conclusion, the findings from this study revealed that *H. pandurifolium* Schrank, *H. foetidum*, *H. petiolare*, *and H. cymocum* are repositories of natural bioactive compounds with potential health-promoting benefits that need to be investigated, for both their antioxidant activity in a number of disease conditions and for further exploration in drug discovery and development projects.

## 1. Introduction

The discovery of natural, safe, and very effective antioxidants has highlighted the need to address health-related problems in recent years. The effectiveness and safety of antioxidant use and the integrity of the body’s antioxidant system are linked to healthy living and the prevention of both life and non-life-threatening diseases. Exogenous or dietary antioxidants work in tandem with the body’s antioxidant system to protect against or combat reactive oxygen species (ROS), also known as free radicals, which cause oxidative stress [1,2]. The overproduction of ROS, such as superoxide anion (O_2_^−^), per hydroxy radical (HOO**^.^**), hydroxyl radical (HO**^.^**) singlet oxygen (^1^O^2^), and hydrogen peroxide (H_2_O_2_), involve consistent or persistent electron reductive pathways to molecular oxygen [3,4]. As a result, chain reactions or processes are created, which cause lipid peroxidation, leading to damage to cell membrane phospholipids, DNA, and protein molecules which are often implicated as oxidative stress inducers in cancer, diabetes mellitus, inflammation, stroke, immunosuppression, anaemia, and neurodegenerative diseases [5].

Antioxidants are chemicals that counteract the imbalances caused by oxidative processes, triggering a defence mechanism against the overproduction of free radicals. Natural, safe, and potent antioxidants that provide defence against the harmful effects or actions of free radicals are gaining immense interest in medical research, as they offer protection against free radicals. Synthetic antioxidants, such as butylated hydroxyanisole (BHA), butylated hydroxytoluene (BHT), and tert-butyl hydroquinone (TBHQ), are linked to high levels of toxicity in humans and are generally expensive [6,7,8,9]. For instance, BHA, a synthetic phenolic antioxidant widely utilised in various sectors, affects endocrine functions, causing significant alterations in oestrogen secretion and steroid hormone homeostasis [10,11,12]. Hence, antioxidants in plant or other natural sources, with few or no adverse effects, are preferred alternatives to synthetic antioxidants, especially because they are affordable and readily available. Fatty acids (FAs) are essential chemical constituents in the cells, which serve as fuel for many biological and metabolic activities, including muscular contraction, and have both nutritional and medicinal values. Medicinal plants are excellent sources of fatty acids in nature and occur in different forms like saturated fatty acids (SFA), monounsaturated fatty acids (MUFA), and polyunsaturated fatty acids (PUFA) [13]. Most of the components of medicinal plants, including fatty acids, terpenes, alkaloids, tannins, terpenoids, saponins, have been shown in various studies to prevent and treat many oxidative stress-related disease conditions [14,15].

The genus *Helichrysum* Mill is a distinctively aromatic medicinal plant of the family Asteraceae, well-distributed in many countries worldwide, including South Africa. *Helichrysum* Mill consists of approximately 600 plant species with at least one-third (245 species) available in South Africa. Many of these species differ in morphology and are therefore classified into 30 different groups. Plants in this genus have been traditionally used for the treatment of such human ailments as cold, cough, skin infections, inflammation, insomnia, cystitis, jaundice, stomach pain, menstrual pain, asthma, arthritis disorders, diabetes mellitus wound healing, etc. The in vitro antioxidant, antifungal, anti-inflammatory, anti-bacterial, hepatoprotective, anti-proliferative, and anti-diabetic properties of some species in this genus have been previously studied [16,17], however the therapeutic potential of *Helichrysum petiolare* & B. L Burtt, *Helichrysum cymocum (L)* D. Don, *Helichrysum foetidum (L).* Moench, *Helichrysum pandurifolium* Schrank as sources of antioxidants, fatty acids, and other constituents has not been fully explored [18]. Only the antioxidant activities of the acetone and methanol extracts of *H. petiolare,* the essential oils from the leaves of *H. cymocum,* and the methanolic extract of *H. foetidum* have been reported in the literature [19,20,21]. Therefore, in this study, GC-MS analysis of the extracts of four selected *Helichrysum* species was done to identify and quantify total phenolic content (TPC), total flavonoids content (TFC), antioxidant activity as well as total fatty acids and lipids.

## 2. Methods

### 2.1. Collection of Plant Material

Four *Helichrysum* species were collected from the Western Cape in the environment of the Cape Peninsula University of Technology (CPUT), Bellville, in October 2020, and samples were identified by Prof. Christopher N. Cupido of the Department of Botany, University of Fort Hare, Alice, South Africa. The accession numbers of the Helichrysum species in this study were: *Helichrysum petiolare*-UFH-2020-10-01, *Helichrysum cymosum*- UFH 2020-10-02, *Helichrysum foetidum*-UFH 2020-10-03, and *Helichrysum pandurifolium*- UFH 2020-10-04.

### 2.2. Plant Extraction

The leaves of the plants were cleaned and air-dried to a constant weight and the dried samples were pulverised using an electronic blender, grounded, and weighed. The powdered plant materials in conical flasks were soaked and subjected to intermittent stirring in 90% aqueous acetone and warmed in the water bath at 60 °C for 2 h with slight modification [22]. The mixture was filtered with Whatman cellulose filter paper under pressure using a pump and the plant material was subjected to a second extraction after soaking overnight and the filtrate pooled before rotary evaporation. The final residue or extract obtained was allowed to dry in the fume cupboard and stored at −20 °C until required for use. (1 mg extract dissolved in 1 ml acetone is used in subsequent analysis).

### 2.3. Chemicals and Reagents

Acetone and other solvents used in this work were purchased from Merck (Darmstadt, Germany), while 1,1-diphenyl-2-picrylhydrazyl (DPPH), ascorbic acid, gallic acid, quercetin, FeCl_3_, AlCl_3,_ and Folin- Ciocalteu reagent were purchased from Sigma chemical Co. (St. Louis, MO, USA). All chemicals used including solvents were of analytical grade.

### 2.4. In Vitro Evaluation

#### DPPH Radical Scavenging Activity Assay

The DPPH radical scavenging activity assay was performed as previously described [23]. Briefly, a 2000 µL stock concentration of DPPH (0.004 g in 100 mL methanol) was added to aliquots of 500 µL plant extracts at different concentrations (10–250 µg/mL) and the reaction mixture was shaken in the dark for 30 min at room temperature. The controls contained the DPPH solution without the plant extract, while methanol was used as the blank. A decrease in absorbance of the test mixture read at 517 nm will result from quenching of DPPH free radicals after the exposure time interval. The following formula is used to determine the scavenging effects of the plant extracts:

% inhibition = [A_0_ − A_1_] × 100/[A_0_] (1)

where A_0_ is the absorbance of the blank and A_1_ is the absorbance of the extract.

### 2.5. Nitric Oxide Scavenging Activity Assay

Sodium nitroprusside generates Nitric oxide (NO) in aqueous physiological pH, measured in the Greiss reaction that produces nitrite ions as previously described [24]. Briefly, 4000 µL of the plant extract or standard solution at different concentrations (10–250 µg/mL) was added to 1000 µL of Sodium nitroprusside solution and 2000 µL of the mixture was added to 1200 µL of the Griess reagent containing 1% sulphanilamide, 0.1% naphthyl ethylenediamine dihydrochloride and 2% H_3_PO_4._ The absorbance of the chromophore formed during diazotisation of nitrite with sulphanilamide, and its subsequent coupling with naphthyl ethylenediamine dihydrochloride, was measured at 550 nm. The percentage (%) inhibition activity was calculated from the following equation, with ascorbic acid as the standard:

% inhibition = [A_0_ − A_1_] × 100/[A_0_] (2)

where A_0_ is the absorbance of the control while A_1_ is the absorbance of the extract or standard.

### 2.6. Reducing Power Assay

Different concentrations of plant extracts (10–250 µg/mL) and corresponding concentrations of standard ascorbic acid were added to 2500 µL and 2500 µL of phosphate buffer (pH6.6) and 1% potassium ferricyanide, respectively. Incubation of the mixture was done at 50 °C for 20 min after which, 2500 µL of 10% trichloroacetic acid was added to the mixture and centrifuged at 3000 rpm for 10 min. Thereafter, 2500 µL of the supernatant was added to 2500 µL distilled water and 500 µL of freshly prepared 0.1% ferric chloride solution [25], and absorbance was read at 700 nm. Ascorbic acid was used as the standard at the various concentrations.

### 2.7. Estimation of Total Phenolic Compounds

The Folin–Ciocalteu reagent method was used to determine the phenolic content as previously described [26]. Briefly, 500 µL of the plant extracts and 100 µL of Folin–Ciocalteu reagent (0.5 N) were added and incubated for 15 min at room temperature after which 2500 µL of sodium carbonate (7.5% *w/v*) was added to the mixture (plant extract + Folin–Ciocalteu) and incubated for 30 min at room temperature, and absorbance read at 760 nm. Phenolic concentration was expressed as gallic acid equivalent (GAE) (mg/g of dry mass) as the reference value.

### 2.8. Total Flavonoid Content Estimation

Aluminium chloride solution was used to determine the flavonoid content as previously described, with quercetin as the standard [27]. Briefly, 1000 µL of a 100 μg/mL extract stock solution was added to 3000 µL of methanol and mixed with 200 µL of 10% aluminium chloride, 200 µL of 1 M potassium acetate, and 5600 µL of distilled water. The mixture was incubated at room temperature for 30 min, and absorbance read at 415 nm. The calibration curve was prepared from quercetin solutions in methanol, at the various concentrations.

### 2.9. Determination of Total Antioxidant Capacity

The plant extract (3000 µL) was added to 3000 µL of the reagent solution containing 0.6 M sulphuric acid, 28 mM sodium phosphate, and 4 mM ammonium molybdate as previously described [28]. The tubes containing the mixture were capped and incubated in the water bath at 95 °C for 90 min and allowed to cool at room temperature, followed by an absorbance reading at 695 nm against the blank.

### 2.10. GC-MS/MS Quantification

#### 2.10.1. Analysis of Phenolic Acids and Phenolic Aldehydes in *H. pandurifolium*, *H. foetidum*, *H. petiolare*, and *H. cymocum* Aqueous Acetone ExtractsSample Preparation

The extraction of 100 mg plant extracts was done for 3 h at 60 °C using 1 ml of 70% methanol, and 130 µL of the extract was freeze-dried and derivatised using 30 µL N, O-Bis (trimethylsilyl)trifluoroacetamide (BSTFA), and 100 µL acetonitrile at 60 °C for 30 min. The sample was then transferred into a 2 mL GC vial, and 1 µL was injected onto the GC-MS/MS in splitless mode.

#### Chromatographic Separation

Helium gas at a flow rate of 1 mL/min, injector temperature maintained at 250 °C, and separation of the analytes was performed on a non-polar Rxi-5Sil MS (30 m, 0.25 mm ID, 0.25 µm film thickness) (instrument type, Trace 1300, Thermo Scientific, Waltham, MA, USA) coupled to triple quadrupole mass spectrometer (TSQ 8000, Thermo Scientific). The oven temperature was programmed as follows: 100 °C for 4 min, then ramped to 180 °C at 10 °C/min rate and held for 2 min before finally ramped at 20 °C/min until 320 °C and held for 5 min. The mass spectrometer detector (MSD) operated in tandem mass spectrometry (MS/MS) mode, the source, and quad temperature were maintained at 250 °C and 150 °C, respectively. The transfer temperature was maintained at 250 °C.

#### 2.10.2. Analysis of Fatty Acid Methyl Esters (FAMEs) in *H. pandurifolium, H. foetidum*, *H. petiolare*, and *H. cymocum* Aqueous Acetone Extracts

##### Sample Preparation

Briefly, 100 mg of the plant extract was vortexed and sonicated at room temperature for 30 min in a mixture of 1 m of chloroform and 1 mL of methanol. This was centrifuged at 3000 rpm for 1 min after which 500 µL of the chloroform fraction (bottom layer) was completely dried with a gentle stream of nitrogen, reconstituted and vortexed with 500 µL of methyl tert-butyl ether (MTBE), and 100 µL was derivatised with 30 µL of trimethyl sulfonium hydroxide (TMSH). Thereafter, 1 µL of the derivatised sample was injected into the GC-MS, in a 5:1 split ratio.

#### Chromatographic Separation

Helium gas at a flow rate of 1.2 mL/min, injector temperature maintained at 240 °C and separation of the FAMEs was performed on a polar RT- 2560 (100 m, 0.25 mm ID, 0.20 µm film thickness) capillary column (instrument type, 6890 N, Agilent technologies network) coupled to Agilent technologies inert XL EI/CI Mass Selective Detector (MSD) (5975, Agilent Technologies Inc., Palo Alto, CA, USA). The oven temperature was programmed as follows: 100 °C for 4 min, then ramped to 240 °C at 3 °C/min rate and held for 10 min. The mass spectrometer detector (MSD) operated in scan mode, and the source and quad temperature were maintained at 250 °C and 150 °C, respectively. The transfer temperature was maintained at 250 °C. The mass spectrometer was operated under electron impact (EI) mode at ionisation energy of 70 Ev, scanning from 40 to 650 *m*/*z*.

### 2.11. Statistical Analysis

The mean ± SD from three experimental observations in triplicates of data were used for statistical analysis. The in vitro antioxidant assays were analysed using the ANOVA test, followed by Tukey’s test, with statistical significance at (*p* < 0.05).

## 3. Results

### 3.1. In Vitro Antioxidant Capacities and Profiles of the Four Helichrysum Species

The antioxidant capacity of the aqueous acetone extracts of four *Helichrysum* species, *namely H. pandurifolium, H. foetidum H. petiolare, and H. cymocum*, were investigated.

### 3.2. DPPH Scavenging Activity

**Figure 1:**
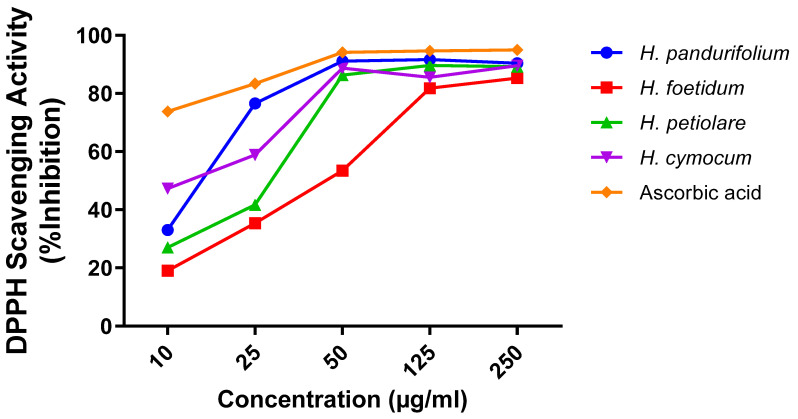
Percentage (%) inhibition of aqueous acetone extract of *H. pandurifolium, H. foetidum H. petiolare, and H. cymocum*.

The radical inhibitory or scavenging activity of the selected aqueous acetone extracts of the Helichrysum species (10–250 µg/mL) was concentration-dependent. Results showed that the extracts caused increased activity with increasing concentrations as shown in Table 1, which is in tandem with results from previous studies [6,29]. Figure 1 shows IC_50_ values of 14.17 ± 1.77 µg/mL *(H. pandurifolium)*, 41.13 ± 3.62 µg/mL *(H. foetidum)*, 23.57 ± 2.59 µg/mL (*H. petiolare)*, and 11.85 ± 3.20 µg/mL *(H. cymocum)* respectively. Hence, the ranking order for the scavenging free radical activity could be represented as *H. cymocum* ˃ *H. pandurifolium* ˃ *H. petiolare* ˃ *H. foetidum* for these extracts, with ascorbic acid used as the standard, showing the best radical scavenging activity and an IC_50_ value of 2.66 µg/mL compared with all four selected *Helichrysum* species.

### 3.3. Nitric Oxide Scavenging Activity

**Figure 2:**
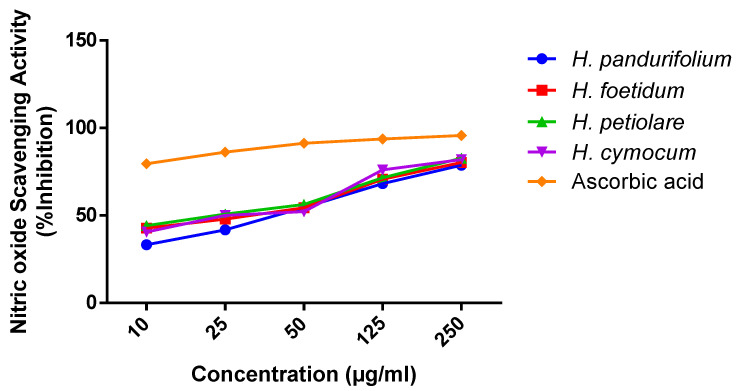
Percentage (%) inhibition of aqueous acetone extract of *H. pandurifolium, H. foetidum H. petiolare, and H. cymocum*.

The scavenging effects of the four Helichrysum species on nitric oxide were concentration-dependent (Table 2) and Figure 2 shows IC_50_ values for the nitric oxide scavenging activity for *H. pandurifolium* (36.19 ± 2.08 µg/mL), *H. foetidum* (24.31 ± 3.67 µg/mL), *H. petiolare* (20.81 ± 3.73 µg/mL), and *H. cymocum* (24.68 ± 4.78 µg/mL), respectively. Thus, the ranking order for the scavenging free radical activity could be represented as *H. petiolare > H. foetidum* > *H. cymocum* ˃ *H. pandurifolium* in scavenging nitric oxide, whereas the ascorbic showed excellent potency of inhibiting nitric oxide with IC_50_ of 0.86 µg/mL.

### 3.4. Reducing Power Activity

**Figure 3:**
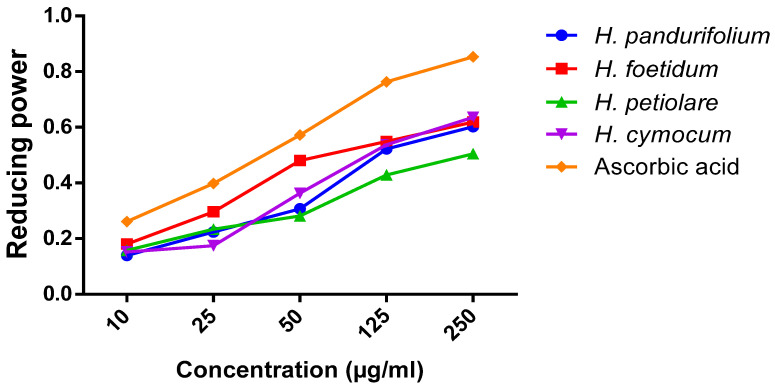
Reducing power activity of aqueous acetone extract of *H. pandurifolium, H. foetidum H. petiolare, and H. cymocum*.

The reducing power dose-response curves of all extracts (10–250 µg/mL) of the selected *Helichrysum* species are concentration-dependent, as shown in Table 3. The ranking order for the reducing power at the highest concentration of 250 µg/mL indicates that *H. cymocum* ˃ *H. foetidum* ˃ *H. pandurifolium* ˃ *H. petiolare* of 0.636 µg/mL, 0.619 µg/mL, 0.602 µg/mL and 0.505 µg/mL, respectively (Figure 3). The ascorbic acid has the highest value of 0.853 µg/mL at the highest concentration of 250 µg/mL.

### 3.5. Total Antioxidant Capacity (TAC), Total Flavonoid (TF), and Total Phenolic (TP) Content

**Figure 4:**
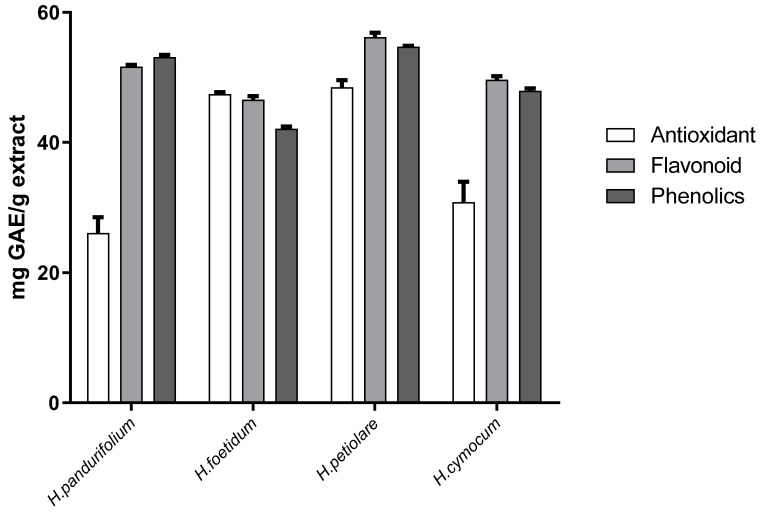
Antioxidant profiles of the aqueous acetone extracts of *H. pandurfolium, H. foetidum, H. petiolare*, *and H. cymocum.* Data is illustrated as mean ± SD (*n* = 3).

Figure 4 shows total antioxidant capacity (TAC) of *H. pandurifolium* (26.11 ± 3.38)*, H. foetidum* (47.44 ± 0.41 mg/g), *H. petiolare* (48.50 ± 1.55 mg/g), and *H. cymocum* (30.82 ± 4.44 mg/g) extracts, respectively, while the flavonoid content was 51.65 ± 0.40 mg/g for *H. pandurifolium*, 46.59 ± 0.75 mg/g for *H. foetidum*, 56.19 ± 1.01 mg/g for *H. petiolare*, and 49.65 ± 0.74 mg/g for *H. cymocum*. On the other hand, the phenolic content was 53.11 ± 0.47 mg/g for *H. pandurifolium*, 42.14 ± 0. 50 mg/g for *H. foetidum*, 54.69 ± 0.23 mg/g for *H. petiolare*, and 47.93 ± 0.57 mg/g for *H. cymocum* acetone extracts, respectively. Overall, *H. petiolare* has the best antioxidant capacity, total flavonoids, and total phenolics compared with other species (Figure 4). Previous studies have shown that many factors, including genetic diversity, biological, environmental, seasonal variations as well as the harvesting period, may account for any differences or similarities seen in the results of TF, TP, and TAC of plant extracts for the same plant species [30,31,32].

### 3.6. Total Phenolic Acid and Phenolic Aldehyde Composition

In Table 4, the composition of the phenolics and their aldehyde content in the four different species vary. The vanillin (78.5 µg/g), protocatechuic acid (297.5 µg/g), coniferaldhyde (13.5 µg/g), and caffeic acid (1424.3 µg/g) were more in the aqueous acetone extract of *H*. *petiolare* in comparison with that of *H. pandurifolium, H. foetidum*, *and H. cymocum*. It was also observed that syringaldehyde (23.1 µg/g), m-coumaric acid (0.392 µg/g), and ferulic acid (144.3 µg/g) were higher in *H. pandurifolium* compared with that of *H.* *petiolare, H. foetidum*, *and H. cymocum*. Additionally, vanillic acid (43.5 µg/g) and p-coumaric acid (0.122 µg/g) showed a higher concentration in *H. foetidum* compared with the other species, meanwhile, syringic acid (13.7 µg/g) and gallic acid (685.7 µg/g) were higher in the aqueous acetone extract of *H. cymocum* in comparison with *H. pandurifolium*, *H.* *petiolare,* and *H. foetidum* as showed in Table 4. Hence, it is important to know that each component was determined using an external standard calibration by GC-MS/MS.

### 3.7. Composition of Saturated Fatty Acids

**Figure 5:**
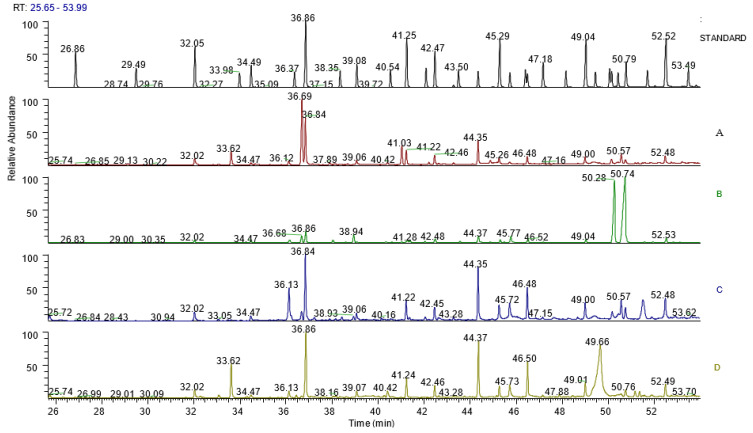
Shows the GC-MS chromatogram of the fatty acids of aqueous acetone extract of *H.pandurifolium, H.foetidum H.petiolare, and H.cymocum* with regularly labelled signals detected by the GC-MS detector. LEGEND: (**A**)—FAMEs chromatogram of *H. pandurifolium;* (**B**)—FAMEs chromatogram of *H. foetidum;* (**C**)—FAMEs chromatogram of *H. petiolare;* (**D**)—FAMEs chromatogram of *H. cymocum*.

Table 5 shows the amount (µg/g) of the individual fatty acid and classes of fatty acids of the aqueous acetone extract of *H. pandurifolium, H. foetidum*, *H. petiolare, and H. cymocum.* Eleven saturated fatty acids (C_12_–C_24_), two monounsaturated fatty acids (C_16_:_1_, C_18_:_1 n 9 (cis)_), and two polyunsaturated fatty acids (C_18_: _2 n 6 (cis)_, C18:3n3) were identified in the extracts of the four plants. The amount of the fatty acids varied widely, viz, 3.1 to 728.3 µg/g (for saturated fatty acids), 76.7 to 1057.9 µg/g (for monounsaturated fatty acids), and 624.9 to 4688.6 µg/g (for polyunsaturated fatty acids), respectively. The PUFA:SFA ratios are 0.604 (*H. pandurifolium*), 0.726 (*H. foetidum*), 0.975 (*H. petiolare*), and 1.202 (*H. cymocum*) with attributable health benefits. Table 6 depicts the GC-MS chromatograms of the fatty acids of aqueous acetone extract of *H.pandurifolium, H.foetidum H.petiolare,* and *H.cymocum* while Figure 5 shows the area of the peaks, ratio area, and the retention time (R, time) of aqueous acetone extract of *H. pandurifolium, H. foetidum H. petiolare, and H. cymocum*.

## 4. Discussion

Antioxidants, fatty acids, and other constituents of medicinal plants have been reported to be beneficial for preventing, alleviating, or treating, oxidative stress-induced diseases [33,34]. Antioxidants are known to be involved in halting redox imbalances by activating the antioxidant defence system to scavenge free radicals through a number of mechanisms, including increased chain-breaking antioxidant activity (synergistic effect), conversion of unstable hydroperoxides in a non–radical pathway to stable components (reducing effect), singlet oxygen (quencher), conversion of pro-oxidant metal derivatives to stable products (metallic chelation), inactivation of pro-oxidant enzymes, decreased activity of free radical oxidation reactions, and inactivation of autoxidation of chain reactions [35,36].

Antioxidants have great therapeutic value as anti-viral, anti-fungal, anti-bacterial, anti-tumoural, anti-cancer, anti-angiogenic, anti-inflammatory, anti-allergic, anti-diabetic, and neuroprotective actions [37,38,39,40]. Flavonoids and phenolics are the main phytocompounds present in most medicinal plants, with more than 4500 flavonoid compounds having been identified [41] and over 8000 phenolic compounds reported [42,43]. Flavonoids and phenolics with potent antioxidant activities have been shown to effectively modulate oxidative stress-related diseases through clearly-defined mechanisms of action [6]. Apart from their medicinal use, antioxidants are also used in the food industry to preserve and improve the shelf-lives of most foods [44].

The determination of the antioxidant potential of plant extracts is dependent on the different methods used and their underlying mechanisms, which explains the multiplicity of techniques in most related studies [45,46]. Therefore, DPPH scavenging activity, nitric oxide scavenging activity, and reducing power activity were used in this study to investigate the antioxidant activities of the aqueous acetone extracts of *H. pandurifolium, H. foetidum H. petiolare, and H. cymocum*. The phenolic antioxidants have been shown to disrupt the formation of ROS and other free radicals by the transfer of hydrogen atoms from its hydroxyl group [47] while the antioxidant flavonoids are known to stabilise ROS via their scavenging actions through the oxidation of free radicals into more stable but less active or reactive radicals [47]. In this study, the aqueous acetone extracts of *H. pandurifolium, H. foetidum, H. petiolare, and H. cymocum* produced radical decolourisation of the DPPH solution because of the high free radical scavenging activity of the plant extracts [5,48].

The results reveal that the extracts tested have a dose-dependent activity. In fact, at the concentration of 250 μg/mL, the aqueous acetone extracts tested reduce the DPPH radical with an excellent percentage of 90.36 ± 1.00%, 85.28 ± 1.34%, 89.18 ± 0.59%, 89.55 ± 1.22%, 89.55 ± 1.22% for aqueous acetone extracts of *H. pandurifolium, H. foetidum H. petiolare, and H. cymocum*, respectively. Additionally, the IC_50_ is inversely proportional to the antioxidant capacity of a compound. However, the lowest value of IC_50_ indicates a strong antioxidant capacity of a compound. *H. Cymocum* showed the lowest IC_50_ values of 11.85 ± 3.20 µg/mL which had better antioxidant activity compared with *H. pandurifolium, H. foetidum*, and *H. petiolare,* (Figure 1). The antioxidant power of the aqueous acetone extracts could be explained by the presence of phenolic compounds including flavonoids present in the species of *Helichrysum* studied and which are known as antioxidant substances with the ability to trap radical species and reactive forms of oxygen. (Figure 1).

The results of the IC_50_ DPPH assay of the methanolic extracts of similar species namely *H. dasyanthum, H. excisum,* and *H. felinum* were 12.33, 13.67, and 20.71 μg/mL, respectively, which were within the range of the IC_50_ obtained in our study, as reported by Lourens et al. [21]. However, only the *H. pandurufolium* of IC_50_ (41.13 ± 3.62 µg/mL) is similar and in agreement with those reported [49] with the species name, *H. chionophilum, H. plicatum subsp. plicatum, and H. arenarium subsp. Aucheri* having IC_50_ of 40.5, 48.0, and 47.6 μg/mL, respectively. From literature, the flavonoids are the main compounds in the helichrysum genus with remarkable antioxidant activity, as reported [49,50].

The reaction of sodium nitroprusside with oxygen produces nitric oxide and nitrite that scavenge free radicals via diazotisation with a sulphanilamide acid coupled reaction, producing a pink colour [51]. The antioxidant activities in NO assay involve the donation of protons to the nitrite radicals that show decreased absorbance. In line with the antioxidant activity, the nitric oxide scavenging revealed dose-dependent activity. It is worth mentioning here that all the doses are highly significant among the groups. Although *H. petiolare* (20.81 ± 3.73 µg/mL) with the lowest IC_50_ indicates the best nitric oxide scavenging effect and good antioxidant compared with the IC_50_ of *H. pandurifolium* (36.19 ± 2.08 µg/mL), *H. foetidum* (24.31 ± 3.67 µg/mL), and *H. cymocum* (24.68 ± 4.78 µg/mL). At the concentration of 250 μg/mL, the aqueous acetone extracts tested have NO scavenging activity with an excellent percentage of 78.64 ± 0.38%, 80.34 ± 0.38%, 82.67 ± 0.58%, 81.98 ± 0.48%, and 89.55 ± 1.22% for aqueous acetone extracts of *H. pandurifolium, H. foetidum H. petiolare, and H. cymocum*, respectively. The difference in the antioxidant of aqueous acetone extracts of *H. pandurifolium, H. foetidum H. petiolare, and H. cymocum* could be attributed to the variation in the chemical composition. Indeed, several types of bioactive compounds known for their antioxidant activity [52,53] are identified in *H. petiolare* with high levels of some compounds, including phenolic (caffeic acid, coniferaldehyde, protocatechuic acid, vanillin) compared to the other species (Table 4). The reducing power of natural products or plant extracts indicates their potential to transfer electrons from Fe^3+^ to Fe^2+^, which is synonymous with the antioxidant activity and is linked to reductones that donate a hydrogen atom to break the free radical chain, thus preventing peroxide formation [54]. The colour change from yellow to various shades of green and blue following treatment is dependent on the reducing power of the plant extract, with the blue colour indicating the highest reducing power. Thus, with increasing concentration of the aqueous acetone extract of *H. pandurifolium, H. foetidum, H. petiolare, and H. cymocum,* the observed blue colour indicates greater reducing power, which is similar to results in previous studies [55,56].

Consequently, the decrease in absorbance observed is an indication of the extent of nitrite radical scavenging potentials [57] and this could be attributed to components such as flavonoids, as reported in previous studies [58,59]. Similarly, the aqueous acetone extracts of *H. pandurifolium*, *H. foetidum* *H. petiolare*, and *H. cymocum* can act as natural antioxidants with relative activities scavenging free radical species. The reducing ability or potential is synonymous with the free radical scavenging activity of the plant extracts which is attributable to different amounts of the plant’s phytochemicals constituents [6] Overall, the antioxidant activities of these plant extracts are attributed to the constituents of total phenolic, total flavonoid, and total antioxidant capacity.

The fatty acid and lipid composition of the aqueous acetone extracts of *H. pandurifolium, H. foetidum, H. petiolare, and H. cymocum* were determined by fatty acid methyl esters (FAMEs) analysis involving the derivatisation, which was analysed by gas chromatography [60]. Previous studies have shown that geographical location, plant species, and seasonal changes could influence the fatty acid content of plants [61,62]. Unsaturated (monounsaturated and polyunsaturated) fatty acids have been reported to ameliorate cardiovascular diseases, modulate inflammation and support the immune system against cancer, diabetes mellitus, neurodegenerative diseases, etc. [63,64]. This study has shown that the aqueous acetone extracts of *H. pandurifolium, H. foetidum H. petiolare, and H. cymocum* contain various amounts of fatty acids with different compositions, as previously reported [61,65]. Our results showed two monounsaturated (MUFA) and two polyunsaturated fatty acids (PUFA), most of which cannot be synthesised by the human body and are only available in dietary sources, making them of great nutritional health benefit [65,66]. Stearic acid (C18:0), oleic acid (C18:1n9 (cis)), and linoleic acid (C18:2n9 (cis)), with known health benefits, were high in the aqueous acetone extracts of *H. pandurifolium, H. foetidum H. petiolare*, and *H. cymocum* as revealed in Table 5, which is similar to findings from previous studies that involved different extractants, different parts, and different *Helichrysum* species, e.g., *H. chionophilum* and *H. plicatum subsp.* [65]. The high dietary fatty acid ratio of PUFA:SFA are implicated in oxidative stress and are prone to lipid peroxidation because PUFA is highly susceptible, however, raising the PUFA/SFA ratio in the body helps to prevent cardiovascular disease (CVD) and conditions [67]. The PUFA/SFA varied considerably in the aqueous acetone extracts of *H. pandurifolium, H. foetidum H. petiolare, and H. cymocum* having 0.604, 0.726, 0.975, and 1.202 (for PUFA/SFA), respectively in our study, and these were seen to be comparable with the values in some seaweed plants considered to be of great health benefits in literature [13]. To the best of our knowledge, no study has reported the comparative study of antioxidant activities, constituents, and fatty acid compositions of four selected aqueous acetone extracts of the *Helichrysum* species. However, few studies have investigated the antioxidant activity of one species of this plant [18]. The many folkloric benefits of the plants in the *Helichrysum* species are under-explored in scientific investigations [18]. Natural, plant-based fatty acids are considered to be the best sources of dietary fatty acids because it has been recommended to prevent cardiovascular (CVD) and other disease conditions [67]. Thus, they could serve as potential sources of effective nutraceutical compounds for the prevention of various disease conditions.

In conclusion, our work provides relevant information on the phenolic, flavonoid, antioxidant capacity, and fatty acid profiles of the aqueous acetone extracts of *H. pandurifolium, H. foetidum H. petiolare, and H. cymocum* which demonstrate significant antioxidant activities. Since these constituents have been reported in previous studies to be effective in the prevention and treatment of various diseases, further research leading to possible drug discovery and development from these four *Helichrysum* species, especially for diabetes and its related cognitive decline conditions, is encouraged.

## Figures and Tables

**Table 1:** DPPH scavenging activity of aqueous acetone extracts of *H. pandurifolium, H. foetidum H. petiolare, and H. cymocum*.

Extract Concentration (µg/mL)	*H. pandurifolium*	*H. foetidum*	*H. petiolare*	*H. cymocum*	Ascorbic Acid
10	33.04 ± 1.78 ^bde^	19.06 ± 3.22 ^acde^	27.08 ± 5.33 ^bde^	47.31 ± 1.41 ^bce^	73.77 ± 5.10 ^abcd^
25	76.53 ± 2.65 ^bcd^	35.39 ± 2.34 ^ade^	41.72 ± 8.80 ^ade^	58.86 ± 6.3 ^abce^	83.38 ± 0.79 ^bcd^
50	91.10 ± 0.67 ^b^	53.50 ± 3.52 ^acde^	86.31 ± 1.81 ^be^	88.67 ± 0.46 ^b^	94.11 ± 0.55 ^bc^
125	91.61 ± 1.11 ^b^	81.83 ± 0.67 ^ace^	89.62 ± 0.45 ^b^	85. 58 ± 2.67 ^e^	94.63 ± 0.67 ^bd^
250	90.36 ± 1.00	85.28 ± 1.34 ^e^	89.18 ± 0.59	89.55 ± 1.22	94.99 ± 0.55 ^b^

Data is presented as a mean ± SD value (*n* = 3); (^a–e^) represents significance (*p* < 0.05) when compared among the groups.

**Table 2:** Nitric oxide scavenging activity of *H. pandurifolium, H. foetidum H. petiolare, and H. cymocum* aqueous acetone extracts.

Extract Concentration (µg/mL)	*H. pandurifolium*	*H. foetidum*	*H. petiolare*	*H. cymocum*	Ascorbic Acid
10	33.27 ± 0.50 ^bcde^	42.85 ± 0.29 ^ade^	44.24 ± 0.50 ^ade^	40.52 ± 0.58 ^abce^	79.58 ± 0.87 ^abcd^
25	41.78 ± 2.05 ^bcde^	47.95 ± 0.66 ^acde^	50.79 ± 1.47 ^abe^	50.09 ±0.38 ^abe^	86.14 ± 0.48 ^abcd^
50	54.51 ± 0.85 ^cde^	54.44 ± 0.38 ^cde^	56.27 ± 0.66 ^bde^	52.17 ± 1.14 ^abce^	91.37 ± 0.61 ^abcd^
125	68.24 ± 0.87 ^bcde^	70.63 ± 0.58 ^ade^	71.52 ± 0.61 ^ade^	76.05 ± 0.29 ^abce^	93.63 ± 0.40 ^abcd^
250	78.64 ± 0.38 ^cde^	80.34 ± 0.38 ^ce^	82.67 ± 0.58 ^abe^	81.98 ± 0.48 ^ae^	95.71 ± 0.40 ^abcd^

Data is presented as a mean ± SD value (*n* = 3); (^a–e^) represents significance (*p* < 0.05) when compared among the groups.

**Table 3:** Reducing power activity of aqueous acetone extracts of *H. pandurifolium, H. foetidum H. petiolare, and H. cymocum*.

Extract Concentration (µg/mL)	*H. pandurifolium*	*H. foetidum*	*H. petiolare*	*H. cymocum*	Ascorbic Acid
10	0.140 ± 0.002 ^bcde^	0.181 ± 0.003 ^acde^	0.158 ± 0.003 ^abe^	0.152 ± 0.003 ^abe^	0.262 ± 0.005 ^abcd^
25	0.224 ± 0.003 ^bd^	0.296 ± 0.006 ^acde^	0.234 ± 0.007 ^bde^	0.174 ± 0.003 ^bce^	0.398 ± 0.005 ^abcd^
50	0.307 ± 0.004 ^bcde^	0.480 ± 0.004 ^acde^	0.282 ± 0.003 ^abde^	0.363 ± 0.004 a^bce^	0.572 ± 0.005 ^abcd^
125	0.522 ± 0.003 ^bcde^	0.549 ± 0.002 ^acde^	0.429 ± 0.005 ^abde^	0.537 ± 0.004 ^abce^	0.763 ± 0.008 ^abcd^
250	0.602 ± 0.007 ^bcde^	0.619 ± 0.005 ^acde^	0.505 ± 0.010 ^abde^	0.636 ± 0.005 ^bce^	0.853 ± 0.008 ^abcd^

Each value represents mean ± SD value (*n* = 3); (^a–e^) indicates significance at (*p* < 0.05) among groups.

**Table 4:** Phenolic acid and phenolic aldehyde composition (µg/g) of the acetone extracts of *H. pandurifolium, H. foetidum, H. petiolare, and H. cymocum*.

TMS Derivative	Mass Spectrum		Plant Extracts (Concentration, µg/g)			
	Pseudomolecular Ions	Fragment Ions	*H. pandurifolium*	*H. foetidum*	*H. petiolare*	*H. cymocum*
Vanillin	224	209	70.1	41.9	78.5	27.5
trans-cinnamic acid	220	205	2.22	2.12	29.6	46.4
Syringaldehyde	254	224	23.1	8.23	14.1	8.11
Vanillic acid	312	282	10.6	43.5	14.8	13.6
Protocatechuic acid	355	311	94.6	84.5	297.5	115.9
Coniferaldehyde	250	235	3.7	1.19	13.5	2.19
m-coumaric acid	293	249	0.392	0.259	0.192	0.237
p-coumaric acid	308	203	0.064	0.122	0.046	0.022
Syringic_Acid	342	312	2.02	4.51	2.95	13.7
Gallic_acid	458	281	31.5	13.7	180.6	685.7
Sinapinaldehyde	280	265	11.2	74.3	18.2	16.9
Ferulic_Acid	338	308	144.3	61.2	98.4	53.7
Caffeic_Acid	396	219	1328.2	928.6	1424.3	1363.2

Legend: the acetone extracts of *H. pandurifolium, H. foetidum, H. petiolare, and H. cymocum* were identified by authentic certified reference materials (CRMs) and compared with calibration standards.

**Table 5:** Composition (µg/g) of saturated fatty acids, monounsaturated fatty acids, polyunsaturated fatty acids, and total fatty acids of the acetone extracts of *H. pandurifolium, H. foetidum H. petiolare*, and *H. cymocum*.

	*SFA*											*MUFA*		*PUFA*									
Concentration (µg/g)	C12:0	C13:0	C14:0	C15:0	C16:0	C17:0	C18:0	C20:0	C21:0	C22:0	C24:0	C16:1	C18:1n9 (cis)	C18:2n6 (cis)	C18:3n3	SFA	MUFA	PUFA	PUFA:SFA	n − 6	n − 3	(n − 6)/(n − 3)	TFA
*H. pandurifolium*	18.6	12.8	451.8	126.3	2446.8	223.1	572.1	240.2	39.0	286.9	166.4	76.7	595.9	2144.1	624.9	4584.0	672.7	2768.9	0.604	2144.1	624.9	3.43	8025.6
*H. foetidum*	62.3	25.3	493.2	349.4	2128.7	160.3	644.7	466.9	83.5	497.6	467.8	92.4	1057.9	2759.6	1147.9	5379.8	1150.3	3907.5	0.726	2759.6	1147.9	2.40	10437.5
*H. petiolare*	22.1	7.9	621.6	318.8	3064.0	261.6	728.3	695.8	100.0	705.7	656.2	286.4	740.3	4688.6	2316.8	7182.0	1026.7	7005.5	0.975	4688.6	2316.8	2.02	15214.2
*H. cymocum*	17.3	3.1	372.0	135.4	2487.8	218.4	563.7	329.3	54.1	439.4	238.0	90.1	516.7	3796.9	2041.3	4858.5	606.8	5838.2	1.202	3796.9	2041.3	1.86	11303.5

SFA—Saturated fatty acids; MUFA—Monounsaturated fatty acids; PUFA—Polyunsaturated fatty acids; TFA—Total fatty acids.

**Table 6:** Shows fatty acids, area of the peaks, ratio area, and the retention time (R, time) obtained from gas chromatography-mass spectrometry (GC- MS) analysis of the aqueous acetone extract of *H. pandurifolium, H. foetidum H. petiolare, and H. cymocum*.

		*H. pandurifolium*			*H. foetidum*			*H. petiolare*			*H. cymocum*		
S/N	Name	R. Time	Area	Area Ratio	R. Time	Area	Area Ratio	R. Time	Area	Area Ratio	R. Time	Area	Area Ratio
1	C12:0	26.83	129390	0.329	26.83	669469	1.043	26.84	120139	0.336	26.84	196681	0.379
2	C13:0	29.48	84201	0.214	29.47	297564	0.464	29.47	55448	0.155	29.49	67203	0.129
3	C14:0	32.02	1908002	4.854	32.02	5051443	7.869	32.02	2077992	5.817	32.02	2843872	5.478
4	C15:0	34.47	492091	1.252	34.47	3564716	5.553	34.47	1036856	2.903	34.47	985986	1.899
5	C16:0	36.84	11057456	28.128	36.86	23592767	36.752	36.84	11014044	30.834	36.86	20445571	39.383
6	C16:1	38.35	206058	0.524	38.34	462768	0.891	38.42	670890	1.878	38.34	462768	0.891
7	C17	39.06	1269355	3.229	39.07	2190159	3.412	39.06	1175330	3.29	39.07	2097968	4.041
8	C18:0	41.22	3120778	7.939	41.28	8058156	12.553	41.22	3107496	8.699	41.24	5352632	10.311
9	C18:1n9c	42.46	2106853	5.359	42.48	8959105	13.956	42.45	2075141	5.809	42.46	3297951	6.353
10	C19:1STD	43.28	393119	N/A	43.33	641949	N/A	43.28	357208	N/A	42.28	519143	N/A
11	C182n6c	44.35	5314280	13.518	44.37	16679594	25.983	44.35	9199072	25.753	44.37	16995730	32.738
12	C20:0	45.26	1337418	3.402	45.31	5943454	9.258	45.26	2891326	8.094	45.27	3170962	6.108
13	C18:3n3	46.48	1700259	4.325	46.52	7547519	11.757	46.48	4938911	13.826	46.5	9919610	19.108
14	C21:0	47.16	187898	0.478	47.19	1035681	1.613	47.15	398686	1.116	47.16	490302	0.944
15	C22:0	49	1673819	4.258	49.04	6573385	10.24	49	3058160	8.561	49.01	4354287	8.387
16	C24	52.48	1797182	4.572	52.53	10192435	15.877	52.48	4709445	13.184	52.49	4136416	7.968

The peaks, ratio area, and the retention time obtained from gas chromatography-mass spectrometry (GC- MS) are described by the National Institute of Standards and Technology (NIST) library to that of a known compound. This is depicted in the different compositions of fatty acids in the above table.

## Data Availability

The data used to support the finding of this study is included in the article.

## References

[1] Sies H. (1997). Oxidative stress: Oxidants and antioxidants. Exp. Physiol. Transl. Integr..

[2] Halliwell B. (2011). Free radicals and antioxidants–quo vadis?. Trends Pharmacol. Sci..

[3] Pryor W.A. (1973). Free radical reactions and their importance in biochemical systems. Federation Proceedings.

[4] Mittler R. (2002). Oxidative stress, antioxidants and stress tolerance. Trends Plant Sci..

[5] Saeed N., Khan M.R., Shabbir M. (2012). Antioxidant activity, total phenolic and total flavonoid contents of whole plant extracts *Torilis leptophylla* L.. BMC Comple. Altern. Med..

[6] Olajuyigbe O.O., Afolayan A.J. (2011). Phenolic content and antioxidant property of the bark extracts of Ziziphus mucronata Willd. subsp. mucronata Willd. BMC Complement. Altern. Med..

[7] Zhao Y., Zhao Q., Lu Q. (2020). Purification, structural analysis, and stability of antioxidant peptides from purple wheat bran. BMC Chem..

[8] Ng K.L., Tan G.H., Khor S.M. (2017). Graphite nanocomposites sensor for multiplex detection of antioxidants in food. Food Chem..

[9] Li Z.-j., Yang F.-j., Yang L., Zu Y.-g. (2018). Comparison of the antioxidant effects of carnosic acid and synthetic antioxidants on tara seed oil. Chem. Cent. J..

[10] Zhao H.-J., Xu J.-K., Yan Z.-H., Ren H.-Q., Zhang Y. (2020). Microplastics enhance the developmental toxicity of synthetic phenolic antioxidants by disturbing the thyroid function and metabolism in developing zebrafish. Environ. Int..

[11] Yang X., Sun Z., Wang W., Zhou Q., Shi G., Wei F., Jiang G. (2018). Developmental toxicity of synthetic phenolic antioxidants to the early life stage of zebrafish. Sci. Total Environ..

[12] Ham J., Lim W., You S., Song G. (2020). Butylated hydroxyanisole induces testicular dysfunction in mouse testis cells by dysregulating calcium homeostasis and stimulating endoplasmic reticulum stress. Sci. Total Environ..

[13] Chen J., Liu H. (2020). Nutritional indices for assessing fatty acids: A mini-review. Int. J. Mol. Sci..

[14] Nwakiban A.P.A., Cicolari S., Piazza S., Gelmini F., Sangiovanni E., Martinelli G., Bossi L., Carpentier-Maguire E., Tchamgoue A.D., Agbor G. (2020). Oxidative stress modulation by cameroonian spice extracts in hepg2 cells: Involvement of nrf2 and improvement of glucose uptake. Metabolites.

[15] Khan M.S.A., Ahmad I. (2019). Herbal medicine: Current trends and future prospects. New Look to Phytomedicine.

[16] Reidel R.V.B., Cioni P.L., Ruffoni B., Cervelli C., Pistelli L. (2017). Aroma profile and essential oil composition of Helichrysum species. Nat. Prod. Commun..

[17] Aslan M., Orhan D.D., Orhan N., Sezik E., Yeşilada E. (2007). A study of antidiabetic and antioxidant effects of Helichrysum graveolens capitulums in streptozotocin-induced diabetic rats. J. Med. Food.

[18] Akinyede K.A., Cupido C.N., Hughes G.D., Oguntibeju O.O., Ekpo O.E. (2021). Medicinal Properties and In Vitro Biological Activities of Selected Helichrysum Species from South Africa: A Review. Plants.

[19] Tirillini B., Menghini L., Leporini L., Scanu N., Marino S., Pintore G. (2013). Antioxidant activity of methanol extract of Helichrysum foetidum Moench. Nat. Prod. Res..

[20] François T., Lambert S.M., Dongmo J., Michel P., Gaby N., Fabrice F., Zache N., Henri A., Chantal M. (2010). Composition, radical scavenging and antifungal activities of essential oils from 3 Helichrysum species growing in Cameroon against Penicillium oxalicum a yam rot fungi. Afr. J. Agric. Res..

[21] Lourens A., Reddy D., Başer K., Viljoen A., Van Vuuren S. (2004). In vitro biological activity and essential oil composition of four indigenous South African Helichrysum species. J. Ethnopharmacol..

[22] Nasr A., Zhou X., Liu T., Yang J., Zhu G.-P. (2019). Acetone-water mixture is a competent solvent to extract phenolics and antioxidants from four organs of Eucalyptus camaldulensis. Turk. J. Biochem..

[23] Chen Y.-X., Liu X.-Y., Xiao Z., Huang Y.-F., Liu B. (2016). Antioxidant activities of polysaccharides obtained from Chlorella pyrenoidosa via different ethanol concentrations. Int. J. Biol. Macromol..

[24] Silva I.K., Soysa P. (2011). Evaluation of phytochemical composition and antioxidant capacity of a decoction containing *Adenanthera pavonina* L. and *Thespesia populnea* L.. Pharmacogn. Mag..

[25] Oraiza M. (1986). Studies on product of browning reaction prepared from glucosamine. Jpn. J. Nutr..

[26] McDonald S., Prenzler P.D., Antolovich M., Robards K. (2001). Phenolic content and antioxidant activity of olive extracts. Food Chem..

[27] Chang C.-C., Yang M.-H., Wen H.-M., Chern J.-C. (2002). Estimation of total flavonoid content in propolis by two complementary colorimetric methods. J. Food Drug Anal..

[28] Prieto P., Pineda M., Aguilar M. (1999). Spectrophotometric quantitation of antioxidant capacity through the formation of a phosphomolybdenum complex: Specific application to the determination of vitamin E. Anal. Biochem..

[29] Motalleb G., Hanachi P., Kua S. (2005). Evaluation of phenolic content and total antioxidant activity in Berberis vulgaris fruit extract. J. Biol. Sci..

[30] Kumar V., Roy B.K. (2018). Population authentication of the traditional medicinal plant Cassia tora L. based on ISSR markers and FTIR analysis. Sci. Rep..

[31] Aryal S., Baniya M.K., Danekhu K., Kunwar P., Gurung R., Koirala N. (2019). Total phenolic content, flavonoid content and antioxidant potential of wild vegetables from Western Nepal. Plants.

[32] Doshi P., Adsule P., Banerjee K. (2006). Phenolic composition and antioxidant activity in grapevine parts and berries (*Vitis vinifera* L.) cv. Kishmish Chornyi (Sharad Seedless) during maturation. Int. J. Food Sci. Technol..

[33] Bland J.S. (1995). Oxidants and antioxidants in clinical medicine: Past, present and future potential. J. Nutr. Environ. Med..

[34] Thomas M.J. (1995). The role of free radicals and antioxidants: How do we know that they are working?. Crit. Rev. Food Sci. Nutr..

[35] Pokorný J. (2007). Are natural antioxidants better–and safer–than synthetic antioxidants?. Eur. J. Lipid Sci. Technol..

[36] Riedl K.M., Lee J.H., Renita M., St Martin S.K., Schwartz S.J., Vodovotz Y. (2007). Isoflavone profiles, phenol content, and antioxidant activity of soybean seeds as influenced by cultivar and growing location in Ohio. J. Sci. Food Agric..

[37] Kotha R.R., Luthria D.L. (2019). Curcumin: Biological, pharmaceutical, nutraceutical, and analytical aspects. Molecules.

[38] Gunathilake K., Ranaweera K., Rupasinghe H. (2018). Change of phenolics, carotenoids, and antioxidant capacity following simulated gastrointestinal digestion and dialysis of selected edible green leaves. Food Chem..

[39] Wang T.-y., Li Q., Bi K.-S. (2018). Bioactive flavonoids in medicinal plants: Structure, activity and biological fate. Asian J. Pharm. Sci..

[40] Tapiero H., Tew K., Ba G.N., Mathe G. (2002). Polyphenols: Do they play a role in the prevention of human pathologies?. Biomed. Pharmacother..

[41] Croteau R., Kutchan T.M., Lewis N.G. (2000). Natural products (secondary metabolites). Biochem. Mol. Biol. Plants.

[42] Tungmunnithum D., Thongboonyou A., Pholboon A., Yangsabai A. (2018). Flavonoids and other phenolic compounds from medicinal plants for pharmaceutical and medical aspects: An overview. Medicines.

[43] Kumar S., Pandey A.K. (2013). Chemistry and biological activities of flavonoids: An overview. Sci. World J..

[44] Leyva-Porras C., Román-Aguirre M., Cruz-Alcantar P., Pérez-Urizar J.T., Saavedra-Leos M.Z. (2021). Application of Antioxidants as an Alternative Improving of Shelf Life in Foods. Polysaccharides.

[45] Frankel E.N., Meyer A.S. (2000). The problems of using one-dimensional methods to evaluate multifunctional food and biological antioxidants. J. Sci. Food Agric..

[46] Nunes X.P., Silva F.S., Almeida J.R.G.d.S., Barbosa Filho J.M., de Lima J.T., de Araújo Ribeiro L.A., Júnior L.J.Q. (2012). Biological Oxidations and Antioxidant Activity of Natural Products.

[47] Kaurinovic B., Vastag D. (2019). Flavonoids and phenolic acids as potential natural antioxidants. Antioxidants.

[48] Krishnaiah D., Sarbatly R., Nithyanandam R. (2011). A review of the antioxidant potential of medicinal plant species. Food Bioprod. Processing.

[49] Tepe B., Sokmen M., Akpulat H.A., Sokmen A. (2005). In vitro antioxidant activities of the methanol extracts of four Helichrysum species from Turkey. Food Chem..

[50] Czinner E., Hagymasi K., Blazovics A., Kery A., Szőke É., Lemberkovics E. (2001). The in vitro effect of Helichrysi flos on microsomal lipid peroxidation. J. Ethnopharmacol..

[51] Balakrishnan N., Panda A., Raj N., Shrivastava A., Prathani R. (2009). The evaluation of nitric oxide scavenging activity of Acalypha indica Linn root. Asian J. Res. Chem..

[52] Sayah K., El Omari N., Kharbach M., Bouyahya A., Kamal R., Marmouzi I., Cherrah Y., Faouzi M.E.A. (2020). Comparative Study of Leaf and Rootstock Aqueous Extracts of Foeniculum vulgare on Chemical Profile and In Vitro Antioxidant and Antihyperglycemic Activities. Adv. Pharmacol. Pharm. Sci..

[53] Zengin G., Sinan K.I., Ak G., Angeloni S., Maggi F., Caprioli G., Kaplan A., Çakılcıoğlu U., Akan H., Jugreet S. (2021). Preliminary investigation on chemical composition and bioactivity of differently obtained extracts from Symphytum aintabicum Hub.-Mor. &Wickens. Biochem. Syst. Ecol..

[54] Govindan P., Muthukrishnan S. (2013). Evaluation of total phenolic content and free radical scavenging activity of Boerhavia erecta. J. Acute Med..

[55] Hazra B., Biswas S., Mandal N. (2008). Antioxidant and free radical scavenging activity of Spondias pinnata. BMC Complementary Altern. Med..

[56] Sharma S., Vig A.P. (2014). Preliminary phytochemical screening and in vitro antioxidant activities of Parkinsonia aculeata Linn. BioMed Res. Int..

[57] Turkoglu A., Duru M.E., Mercan N., Kivrak I., Gezer K. (2007). Antioxidant and antimicrobial activities of Laetiporus sulphureus (Bull.) Murrill. Food Chem..

[58] Lakhanpal P., Rai D.K. (2007). Quercetin: A versatile flavonoid. Internet J. Med. Update.

[59] Boora F., Chirisa E., Mukanganyama S. (2014). Evaluation of nitrite radical scavenging properties of selected Zimbabwean plant extracts and their phytoconstituents. J. Food Processing.

[60] Eder K. (1995). Gas chromatographic analysis of fatty acid methyl esters. J. Chromatogr. B: Biomed. Sci. Appl..

[61] Foseid L., Devle H., Stenstrøm Y., Naess-Andresen C.F., Ekeberg D. (2017). Fatty acid profiles of stipe and blade from the Norwegian brown macroalgae Laminaria hyperborea with special reference to acyl glycerides, polar lipids, and free fatty acids. J. Lipids.

[62] Schmid M., Stengel D.B. (2015). Intra-thallus differentiation of fatty acid and pigment profiles in some temperate Fucales and Laminariales. J. Phycol..

[63] Ahmad S., Ahmad S., Bibi A., Ishaq M.S., Afridi M.S., Kanwal F., Zakir M., Fatima F. (2014). Phytochemical analysis, antioxidant activity, fatty acids composition, and functional group analysis of Heliotropium bacciferum. Sci. World J..

[64] Calder P. (1999). Dietary fatty acids and the immune system. Lipids.

[65] Acet T., Ozcan K., Zengin G. (2020). An assessment of phenolic profiles, fatty acid compositions, and biological activities of two Helichrysum species: H. plicatum and H. chionophilum. J. Food Biochem..

[66] Sabudak T., Ozturk M., Goren A.C., Kolak U., Topcu G. (2009). Fatty acids and other lipid composition of five Trifolium species with antioxidant activity. Pharm. Biol..

[67] Kang M.J., Shin M.S., Park J.N., Lee S.S. (2005). The effects of polyunsaturated: Saturated fatty acids ratios and peroxidisability index values of dietary fats on serum lipid profiles and hepatic enzyme activities in rats. Br. J. Nutr..

